# Anti-inflammatory effect of *Adiantum* *capillus-veneris* hydroalcoholic and aqueous extracts on acetic acid-induced colitis in rats

**Published:** 2020

**Authors:** Ladan Khoramian, Seyed-Ebrahim Sajjadi, Mohsen Minaiyan

**Affiliations:** 1 *School of Pharmacy and Pharmaceutical Sciences, Isfahan University of Medical Sciences, Isfahan, Iran*; 2 *Department of Pharmacognosy, School of Pharmacy and Pharmaceutical Sciences, Isfahan University of Medical Sciences, Isfahan, Iran*; 3 *Department of Pharmacology and Toxicology and Isfahan Pharmaceutical Sciences Research Center, School of Pharmacy and Pharmaceutical Sciences, Isfahan University of Medical Sciences, Isfahan, Iran*

**Keywords:** Adiantum capillus-veneris, Colitis, Inflammation, Plant extract, Animal model

## Abstract

**Objective:**

Colitis is an inflammatory bowel disease with unknown etiology where many factors might play a role. * Adiantum capillus-veneris *may have beneficial effects in colitis because of its anti-inflammatory, antioxidant, wound healing and antimicrobial effects. The aim of this study was to explore the anti-inflammatory and anti-ulcerative effects of *A. capillus-veneris *on acetic acid-induced colitis in a rat model.

**Materials and Methods:**

*A. capillus-veneris* aqueous (ACAE; 150, 300, and 600 mg/kg) and hydroalcoholic extract (ACHE; 150, 300, and 600 mg/kg) were given orally (p.o.) to male Wistar rats 2 hr before induction of colitis by intra-rectal administration of acetic acid 3%, and continued for 4 days. Prednisolone (4 mg/kg) and mesalazine (100 mg/kg) were applied p.o., as reference drugs for comparison. On day five, colitis indices of tissue specimens were evaluated and levels of biochemical markers including myeloperoxidase (MPO) and malondialdehyde (MDA) were determined.

**Results:**

In all groups treated with ACAE and ACHE with the exception of ACAE (150 mg/kg), ulcer index and wet weight of colon as parameters of macroscopic injuries, total colitis index as marker of microscopic features and MPO activity were significantly reduced in comparison to the control group; however, MDA value was only diminished in ACAE (300 and 600 mg/kg) and ACHE (300 mg/kg) groups significantly.

**Conclusion:**

This research showed that ACAE and ACHE had dose-related beneficial effects on acetic acid-induced colitis and these effects could be attributed to anti-inflammatory, ulcer healing and antioxidant activities of these extracts.

## Introduction

The idiopathic inflammatory bowel disease (IBD) is a prevalent gastrointestinal inflammatory condition which is divided into ulcerative colitis and Crohn's disease (Baumgart and Carding, 2007 ▶; Head and Jurenka, 2003 ▶). IBD is a multifunctional disorder and its etiology is unknown but one or more of the following factors such as immune system dysfunction (caused by genetic or environmental factors), changes in normal flora of the gastrointestinal tract, mucosal barrier damage and oxidative stress play a pivotal role in this disorder (Freidman et al., 2015 ▶).

Current treatments for IBD are 5-amino-salicylic acid derivatives such as sulfasalazine and mesalazine that have anti-inflammatory effects, corticosteroids, immunomodulatory drugs including azathioprine, 6-mercaptopurine and methotrexate, and antitumor necrosis factor-alpha (TNFα) such as infliximab and adalimumab (Burger and Travis, 2011 ▶; Mcquaid et al., 2018 ▶).

Sulfasalazine has some adverse effects such as rash, agranulocytosis, pancreatitis and hepatitis. Corticosteroids increase the risk of osteoporosis and immune system suppression. Azathioprine and 6-mercaptopurine exert serious adverse reactions such as pancreatitis, blood disorders and hepatotoxicity while methotrexate can cause pulmonary fibrosis, myelosuppression and hepatotoxicity (Schwab et al., 2002 ▶; Rogler, 2010 ▶; Mcquaid et al., 2018 ▶).

Because of the adverse effects of these drugs and unclear etiology of ulcerative colitis, it is highly required to search for alternative treatments among herbal and traditional medicines (Rahimi et al., 2010 ▶; Wan et al., 2014 ▶). 

*Adiantum capillus-veneris (A. capillus-veneris*) from Adiantaceae family is named “*Pare-siavoshan”* in Iranian Traditional Medicine (ITM) and is one of the common and widely distributed plants in the world. It contains flavonoids (*e.g.* rutin, quercetin, isoquercetin, astragalin and kaempferol), phenylpropanoids, triterpenoids, carbohydrates, carotenoids, mucilage and many other components (Dehdari et al., 2018 ▶). In ITM, it is used for alleviation of chest complaint, as a cold and cough remedy, antidandruff and hair tonic, and to increase lactation, and improve kidney function, jaundice and hepatitis (Al-Snafi, 2015 ▶; Haider et al., 2011 ▶; Ghasemi-Dehkordi, 2002 ▶). 

*A. capillus-veneris* has shown wound healing effects *in vitro* likely due to its angiogenic and antioxidant properties that are attributed to its polar and flavonoid components such as quercetin, kaempferol and rutin (Askari et al., 2012 ▶; Calderon-Montano et al., 2011 ▶; Nilforoushzadeh et al., 2014 ▶). Topical application of *A. capillus-veneris* leaves for the treatment of open wounds has been recommended in Qrabadian-e Kabir, written by Aghili Khorasani. Negahdari et al. (2017) ▶ mentioned that *A. capillus-veneris* ethanol extract was effective in wound healing likely through the regulation of TGF-β 1 and VEGF-A gene expression in fibroblasts. This activity was also affirmed by Nilforoushzadeh et al. (2014) ▶ in an experimental *in vitro* study carried out on human dermal fibroblasts and human umbilical vein endothelial cells. The researchers proposed that the plant extract might be useful in prevention of late radiation-induced injuries after radiation therapy and for healing of external wounds like burns and bedsores. Ethanol extract of *A.* *capillus-veneris* and its fractions were reported to possess anti-inflammatory effects in animal models by suppressing nitric oxide (NO), interleukin-6 (IL-6) and tumor necrosis factor alpha (TNF-α) synthesis and release as well as inhibiting nuclear factor kappa-b (NF-κB) activation (Haider et al., 2011 ▶; Yuan et al., 2013 ▶). Researches have shown that * A. capillus-veneris *leaves extract are rich in radical scavenging molecules like terpenoids, flavonoids, saponins and tannins (Rajurkar and Gaikwad, 2012 ▶). Moreover, alcoholic extract of *A. capillus-veneris* was reported to have antidiabetic effects in a rabbit model because of its flavonoids and antioxidant content (Ibraheim et al., 2011 ▶).

Taken together, it is assumed that* A. capillus-veneris *may have beneficial effects in IBD because of its anti-inflammatory, antioxidant, radical scavenging and wound healing properties. So, in the present study, the effect of *A. capillus-veneris *hydroalcoholic (ACHE) and aqueous extract (ACAE) was evaluated on acetic acid-induced colitis in rats.

## Materials and Methods

**Chemicals and drugs**

Prednisolone and mesalazine powders were purchased from Iran Hormone Co. (Tehran, Iran). Ortho-dianisidine dihydrochloride (ODD) and hexadecyl trimethyl ammonium bromide (HTAB) reagents were obtained from Sigma Company (St. Louis, USA). Formaldehyde, glacial acetic acid, ethanol (96%) and diethyl ether oxide were purchased from Merck Company (Darmstadt, Germany). Normal saline was purchased from Shahid Ghazi Co. (Tehran, Iran).

**Plant preparation**

*A. capillus-veneris *was purchased from a local traditional market in Yazd, Iran and was identified by a botanist Dr. Iraj Mehregan from Islamic Azad University, Tehran Branch and a herbarium voucher (No. 2460) was deposited at Pharmacognosy Department of Isfahan School of Pharmacy. Aerial parts of the plant were dried in shade and finely powdered. Aqueous extract was prepared by maceration method using 4 l distilled water for 300 g plant powder, and the mixture was left for 24 hr; then, it was shaken for 2 hr, finally filtered and dried by a rotary evaporator. For preparation of hydroalcoholic extract, 300 g of plant powder was macerated by 3 l of EtOH/H_2_O (80/20) for 24 hr and then shaken for 2 hr, filtered and evaporated by a rotary evaporator. This semisolid extract was dried by a freeze drier for more complete drying (Evans, 2009 ▶). 

**Animals**

Sixty male Wistar rats (180-220 g) were purchased from animal house of Isfahan School of Pharmacy, Isfahan University of Medical Science, Isfahan, Iran. All the animals were kept in the same and standard environmental situation. They were fed with pelleted rat chow and tap water, *ad libitum*. The study was approved by the Animal Research Ethics Committee of Isfahan University of Medical Sciences in Iran (ethics approval code: IR.MUI.RESEARCH.REC.1397.154) and performed in accordance with National Institute of Health Guide for the Care and Use of Laboratory Animals. Maximum efforts were made to decrease the number of animals used in the study and to lower the experimental distress.

**Animal grouping**

Ten groups of 6 rats (at least) were used in this study as follows:

1: Sham group (normal group): received normal saline/tween orally (5 ml/kg, p.o.), as vehicle 2 hr before intra-rectal injection of normal saline (2 ml/rat).

2: Control (colitis) group: received normal saline/tween (5 ml/kg, p.o.) as vehicle, 2 hr before induction of colitis and continued for 4 days.

3, 4, and 5: Aqueous extract groups: received 3 increasing doses of ACAE (150, 300, and 600 mg/kg), 2 hr before induction of colitis and continued for 4 days (Kasabri et al. 2017).

6, 7, and 8: Hydroalcoholic extract groups: received 3 increasing doses of ACHE (150, 300, and 600 mg/kg) 2 hr before induction of colitis and continued for 4 days.

9. Prednisolone group (reference group): received prednisolone (4 mg/kg, p.o.), 2 hr before induction of colitis and continued for 4 days.

10: Mesalazine group (reference group): received mesalazine (100 mg/kg, p.o.), 2 hr before induction of colitis and continued for 4 days (Heidari, 2016)

All examined doses of plant extracts were dispersed in normal saline containing tween 80 (0.1%), and administered p.o. in uniform volume (2 ml/rat).

**Induction of ulcerative colitis**

For induction of colitis, the rats were fasted for 24 hr while water was freely available. Then, under light ether anesthesia, 2 ml of acetic acid 3% was instilled within the colon via a flexible catheter (2 mm diameter and 8 cm length). The animals were maintained in head down position for one minute to prevent anal leakage (Minaiyan et al., 2011 ▶).

**Preparation of colon tissue for macroscopic and microscopic studies**

Twenty-four hours after giving the last dose of extracts, the rats were euthanized via over-dose of carbon dioxide inhalation. Distal colons (8 cm in length and 3 cm above the anus) were removed, cut longitudinally, and washed with normal saline and their wet weight was immediately measured. Then, the tissue was fixed on a white working board and some suitable photos were taken by IPhone 8 camera for subsequent macroscopic evaluation. Ulcer severity (US) was evaluated through following scores: 0: No ulcer or erosion, 1: inflammation, edema, thickness and superficial erosions, 2: hemorrhage and evident erosion, and 3: severe ulceration, tissue necrosis and even perforation. Ulcer area (UA) was measured by Fiji P Win 32 program using photos of colons. Ulcer index (UI) was obtained through following equation: UI = UA (cm^2^) + US (1-3) (Minaiyan et al., 2014 ▶; Latifi et al., 2015 ▶).

After macroscopic evaluations, each colon specimen was cut into 2 equal pieces along its length. One piece was kept in formalin 10% for microscopic and histopathologic assessment while the other piece was kept in freezer (-70°C) for myeloperoxidase (MPO) and malondialdehyde (MDA) activity measurements (Minaiyan et al., 2009 ▶).

For microscopic studies, fixed colonic tissue specimens were paraffin-embedded, processed and sectioned in 4 millimeter (mm) thick layers. Then, they were deparaffinized by xylene and hydrated by ethanol. Finally, they were stained with hematoxylin and eosin (H&E). Inflammation severity (0: none, 1: slight, 2: moderate, and 3: severe), inflammation extent (0: none, 1: mucosal, 2: mucosal and submucosal, and 3: transmural invasion), crypt damage (0: none, 1: basal 1/3 damaged, 2: basal 2/3 damaged, 3: surface epithelium was intact only, and 4: crypts and surface epithelium were intact) and leukocyte infiltration (0: trace, 1: mild, 2: moderated, and 3: sever) were assessed in H&E-stained encoded sections following modification of a validated scoring scheme described by Cooper et al. (1993) ▶. Total colitis index (TCI) was measured by the following formula: TCI = Inflammation severity + inflammation extent + crypt damage + leukocyte infiltration. Histopathological evaluation was carried out using a Zeiss microscope equipped with a Sony color video camera (Sony, Japan) for digital imaging.

**Evaluation of MPO activity**

 For measuring the MPO activity of colonic tissue, 100 mg of each colon specimen was homogenized in 5 ml of potassium buffer (pH 6) containing 0.5% HTAB for three 45-sec cycles. The homogenates were sonicated for 10 sec in an ice bath, and then, they were centrifuged at 4000 rpm for 15 min. 0.1 ml of supernatants was mixed with 2.9 ml of 50 mM phosphate buffer (pH 6) containing 0.167 mg/ml ODD and 0.0005% hydrogen peroxide. Finally, the absorbance (at 450 nm) was measured 0 and 3 minutes after that by a UV-Vis spectrophotometer (LSI Model Alfa-1502). MPO activity is reported as U/100 mg weight of wet colon tissue (Motavallian-Naeini et al., 2012 ▶).

**Evaluation of MDA content**

For MDA activity measurement, 1 ml of potassium chloride 1.15% w/v was added to 100 mg of chopped colon tissue. The mixture was homogenized and then, centrifuged at 12000 rpm for 10 min. The supernatant was separated and used for measuring MDA value by an assay kit (Navand-Salamat, Iran) according to its provider’s instructions. MDA content is reported as nmol/ml of tissue sample.

**Statistical analysis**

Statistical analyses were performed using SPSS software version 23. Parametric data is expressed as mean±SEM, and compared using one-way ANOVA with Tukey's LSD *post hoc* test. Non parametric data is expressed as median (range) using Mann-Whitney U-test for analysis. A p<0.05 was considered significant difference level for all analyses.

## Results

**Extract characterization**

The yield values for aqueous and hydroalcoholic extracts were obtained 15.7 and 10.7% w/w, respectively. 

**Effect of ACAE and ACHE on macroscopic parameters**

In the sham group, there were no changes in colonic architecture suggesting that handling and surgical procedure had no interference with the results of the current experiment. 

**Figure 1:**
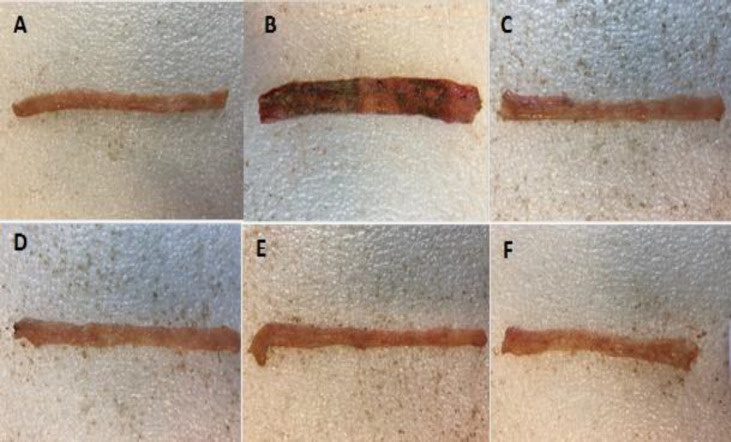
Photos of colon tissue, 5 days after acetic acid-induced colitis in rats: (A) Normal colon treated with normal saline/tween (Sham); (B) Control colitis treated with normal saline/tween; (C) Colitis treated with ACAE (600 mg/kg); (D) Colitis treated with ACHE (300 mg/kg); (E) Colitis treated with prednisolone (4 mg/kg); (F) Colitis treated with mesalazine (100 mg/kg)

In all groups treated with ACAE and ACHE extracts, but with the exception of ACAE (150 mg/kg) group, ulcer score and area, ulcer index and weight of wet colons (8 cm in length) were reduced significantly compared to the untreated control group (at least p<0.05) (Table 1 and Figure 1). In groups treated with prednisolone and mesalazine as reference drugs, ulcer score, ulcer area (cm^2^), ulcer index and wet weight of colon (mg/cm) were significantly reduced in comparison with the control group (at least p<0.001).

**Table1:** Effect of ACAE and ACHE on the macroscopic parameters of colitis induced by acetic acid in rats

Group/dose (mg/kg)	Ulcer area (cm^2^)	Ulcer score (0-3)	Ulcer index (0-11)	Colon weight (mg/cm)
Sham	0.00±0.00	0.0 (0-0)	0.00±0.00	79.8±5.1
Control (Colitis)	5.95±0.43+++	3.0 (3-3)+++	8.95±0.42+++	250.6±7.4+++
ACAE150	4.01±0.65	3.0 (1-3)	6.51±0.83	229.1±18.6
ACAE300	2.05±0.58^***^	1.0 (1-2)^**^	3.22±0.74^***^	159.4±12.5^***^
ACAE600	1.68±0.33^***^	1.0 (1-2)^**^	3.01±0.53^***^	118.7±7.3^***^
ACHA150	3.43±1.05^*^	1.5 (1-3)^*^	5.27±1.34^*^	146.4±19.5^***^
ACHA300	1.36±0.43^***^	1.0 (0-3)^**^	2.53±0.70^***^	104.1±9.9^***^
ACHA600	3.22±0.69^*^	2.0 (1-3)^**^	5.06±0.97^**^	150.0±9.2^***^
Prednisolone4	0.53±0.05^***^	1.0 (1-1)^***^	1.53±0.05^***^	89.6±2.2^***^
Mesalazine100	0.62±0.05^***^	1.0 (1-2)^***^	1.79±0.18^***^	108.8±10.0^***^

Sham: normal rats received normal saline/tween (5 ml/kg/day), Control: rats with colitis received normal saline/tween (5 ml/kg/day), ACAE: *A. capillus-veneris* aqueous extract (150, 300 and 600 mg/kg), ACHE: *A. capillus-veneris* hydroalcoholic extract (150, 300 and 600 mg/kg). Data is expressed as mean±SEM or median (range) for scoring parameter, n=6. +++p<0.001 denotes a significant difference versus sham group. ^*^p<0.05, ^**^p<0.01, and ^***^p<0.001, denote a significant difference versus control.

**Effect of ACHE on microscopic parameters**

**Figure 2:**
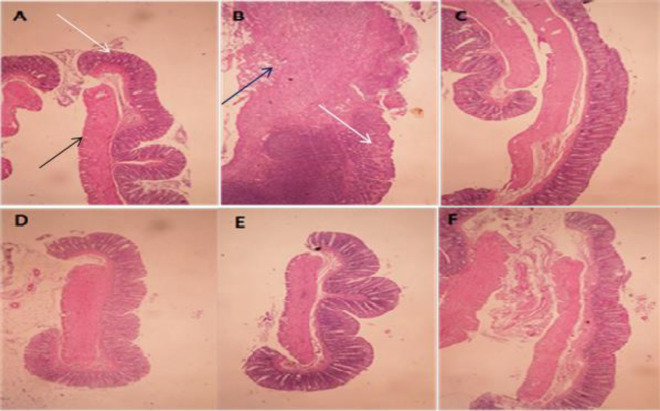
Microscopic illustration of colonic tissue in rats. (A) Normal tissue, treated with normal saline/tween (5 ml/kg); (B) Colitis control group tissue which shows crypt damage (white arrows), leucocytes infiltration, mucus and sub-mucosal layer edema and inflammation (black arrows); (C) Colitis treated with ACAE (600 mg/kg); (D) Colitis treated with ACHE (300 mg/kg); (E) Colitis treated with prednisolone (4 mg/kg); (F) Colitis treated with mesalazine (100 mg/kg). H&E staining at x10 magnification (panel B was x40 magnified).

As it was expected, tissue specimens were intact and healthy in the normal group. In the colitis control group, however, edema, inflammation, hemorrhage, crypt damage and vast leukocyte infiltration were found as the most severe alterations evident in colon tissue (Figure 2). 

 All doses of ACHE and ACAE fractions reduced total colitis index compared to the control group (at least p<0.5), albeit in group which received ACAE 150 mg/kg, ameliorations of pathologic parameters were not significant (p>0.05). In reference groups that received prednisolone or mesalazine, these pathological features were significantly improved compared to the control group (at least p<0.05) (Table 2).

**Table 2:** Effect of ACAE and ACHE on the microscopic parameters of colitis induced by acetic acid in rats

Group/dose (mg/kg)	Inflammation severity (0-3)	Inflammation extent (0-3)	Leukocyte infiltration (0-3)	Crypt damage (0-4)	Total colitis index (0-13)
Sham	0.0 (0-0)	0.0 (0-0)	0.0 (0-0)	0.0 (0-0)	0.0 (0-0)
Control (colitis)	3.0 (3-3)+++	3.0 (2-3)+++	3.0 (2-3)+++	3.5 (3-4)+++	12.5 (11-13)+++
ACAE150	2.0 (1-3)^*^	2.0 (1-3)	2.5 (2-3)	3.0 (3-4)	9.5 (7-13)
ACAE300	1.0 (0-1)^**^	1.5 (0-2)^*^	1.5 (1-2)^*^	1.5 (1-2)^**^	5.5 (3-7)^**^
ACAE600	1.0 (0-2)^***^	1.0 (0-2)^***^	1.0 (1-2)^***^	1.0 (1-2)^***^	4.5 (2-8)^***^
ACHE150	2.5 (1-3)	2.0 (1-3)	2.0 (1-3)^*^	2.5 (1-4)	9.0 (4-12)^*^
ACHE300	1.0 (0-3)^***^	1.5 (0-1)^***^	1.0 (0-2)^***^	1.0 (1-3)^***^	4.5 (1-9)^***^
ACHE600	2.0 (2-3)^**^	2.0 (1-3)	2.5 (1-3)	2.5 (2-3)^*^	9.0 (6-11)^**^
Prednisolone4	1.0 (0-1)^***^	0.5 (0-1)^***^	1.0 (0-1)^***^	0.0 (0-2)^***^	2.5 (2-3) ^***^
Mesalazine100	0.5 (0-1)^***^	0.5 (0-1)^***^	1.0 (0-1)^***^	1.0 (0-2)^***^	3.0 (0-5)^***^

Sham: normal rats received normal saline/tween (5 ml/kg/day), Control: rats with colitis received normal saline/tween (5 ml/kg/day), ACAE: *A. capillus-veneris *aqueous extract (150, 300 and 600 mg/kg), ACHE: *A. capillus-veneris *hydroalcoholic extract (150, 300 and 600 mg/kg). Data is expressed as median (range) for scoring parameter, n=6. +++p<0.001 denotes a significant difference versus the sham group. ^*^ p<0.05, ^**^p<0.01, and ^***^p<0.001, denote a significant difference versus control.

**Myeloperoxidase activity measurements**

**Figure 3:**
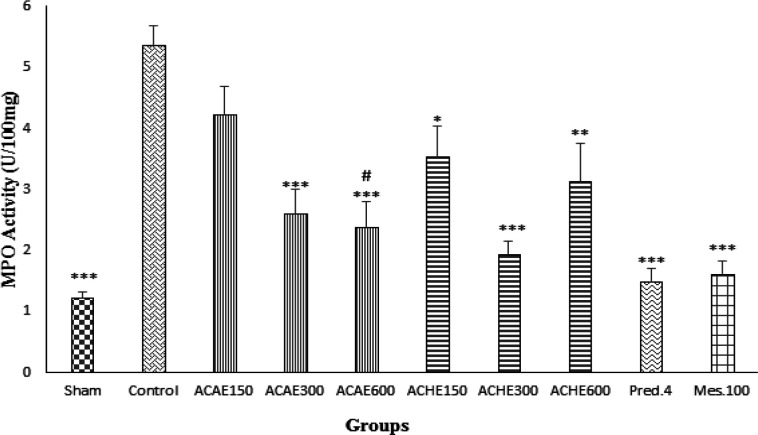
Myeloperoxidase (MPO) activity in colonic tissue of rats. Sham and control colitis treated with normal saline/tween (5 ml/kg), *A. capillus-veneris* aqueous extract (ACAE; 150, 300, and 600 mg/kg), *A. capillus-veneris* hydroalcoholic extract (ACHE; 150, 300, and 600 mg/kg), prednisolone (Pred., 4 mg/kg) and mesalazine (Mes, 100 mg/kg). Data is presented as mean±SEM. *p<0.05, **, p<0.01, and ***p<0.001, denote a significant difference versus control. # p<0.05 denotes a significant difference versus ACAE150 mg/kg

Results of this part of the study showed that MPO activity in groups treated with ACAE 300 and 600 mg/kg and ACHE (150, 300, and 600 mg/kg) was diminished significantly (at least p<0.05) in comparison with untreated control group (Figure 3). Besides, prednisolone and mesalazine reduced MPO activity in reference groups (p<0.001); however, MPO activity reduction in ACAE 150 mg/kg group was not meaningful (p>0.05).

**Malondialdehyde value measurements**

**Figure 4:**
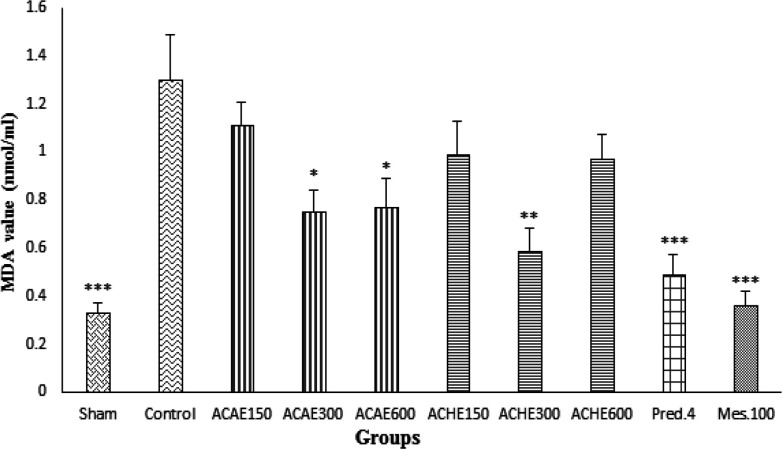
Malondialdehyde (MDA) values in colonic tissue of rats. Sham and control colitis treated with normal saline/tween (5 ml/kg), *A. capillus-veneris* aqueous extract (ACAE), *A. capillus-veneris* hydroalcoholic extract (ACHE), prednisolone (Pred., 4 mg/kg) and mesalazine (Mes, 100 mg/kg). Data is presented as mean±SEM. *p<0.05, **p<0.01, and ***p<0.001, denote a significant difference versus control

MDA (thiobarbituric acid reactants) value decreased in all groups treated with ACAE and ACHE compared to the control group although this fall was just significant for ACAE (300 and 600 mg/kg) and ACHE (300 mg/kg) (at least p<0.05). Other extract treated groups did not express significant alterations in MDA value. Moreover, in groups that received prednisolone and mesalazine as reference drugs, MDA decline was significant in treated animals (p<0.001) (Figure 4).

## Discussion

In the present study, according to macroscopic, microscopic and biochemical evaluations, ACAE and ACHE had protective effects against acetic acid-induced colitis in rats. Our results showed significant increases in colonic weight, severe ulceration and even necrosis of colon tissue in the control group after applying acetic acid as an established method for induction of ulcerative colitis (Al-Rejaie et al., 2013 ▶). Besides, our results indicated that MPO and MDA values of colonic tissue increased in the untreated control group compared to the normal group suggesting macrophage and neutrophil activation and migration which in turn, resulted in increased oxidative stress (Yao et al., 2010 ▶). According to our investigation, all doses of ACAE and ACHE, with the exception of ACAE (150 mg/kg), could alleviate colitis indices by considering markers of macroscopic, microscopic and biochemical injuries in colonic tissue. Moreover, according to our results, MPO activity of all groups with the exception of ACAE (150 mg/kg), and MDA levels in ACHE (300 and 600 mg/kg) and ACHE (300 mg/kg) groups were significantly attenuated. It is presumed that reduction of colitis parameters in group that received ACHE (300 mg/kg) was greater than other groups such as ACHE 600 mg/kg though the difference was not significant. It is assumed that some harmful ingredients might exist in the hydroalcoholic extract which can counteract with its beneficial activity on colitis at higher doses (Ahmed et al., 2011 ▶; Minaiyan et al., 2014 ▶). Complete toxicity evaluations are highly recommended for both extracts although there are some reports that verified its safety both *in vivo* and *in vitro* (Dehdari et al., 2018 ▶). For this purpose, acute oral toxicity studies of the aqueous and methanol extracts were executed in rats. An acute single dose of 2000 mg/kg was administered to rats and after 30 min, and 4 and 24 hr, main changes in behavior and death were evaluated. Both extracts displayed no major changes in behavior as well as lethality rate after elapsed times (Ranjan et al., 2014 ▶). Albeit, it is recommended that the plant should not be used during lactation and pregnancy period because of no available and compelling human data (Gruenwald J et al., 2008 ▶).

On the other hand, we did not see healing effects for ACAE 150 mg/kg, probably because not enough active ingredients exist in the aqueous extract at this low dose or they were not available via oral administration and it is required to try other drug delivery methods such as parenteral or rectal routes of administration for the aqueous extract.

 According to Yuan et al. ethanol extract of *A. capillus-veneris *had anti-inflammatory effect through inhibiting NF-κB activation and suppressing the production of inflammatory mediators such as NO, IL-6, TNFα and LPS-induced PGE2 which are important in IBD pathology. They accounted phenolic compounds, flavonoids and triterpenoids as ingredients inducing these effects (Yuan et al., 2013 ▶; Ibraheim et al., 2011 ▶; Dehdari and Hajimehdipoor, 2018 ▶). Moreover, this extract showed anti-edema and antinociceptive effects similar to non-steroidal anti-inflammatory drugs (NSAIDs), however, it did not cause any gastric ulceration compared to ibuprofen used as control (Haider et al., 2011 ▶). Rajurkar et al. (2012) ▶ reported that aqueous extract of *A. capillus-veneris* had the highest amounts of phenolic compounds compared to methanol and ethanol extracts whereas in our study, the best results were obtained for the ethanol extract of *A. capillus-veneris*. This controversy may reveal that some active ingredients in the ethanol extract like triterpenoids are not among phenolic compounds which are responsible for inhibiting cyclooxygenase pathway (Haider et al., 2011 ▶; Haider et al., 2013 ▶). We know that phenolic compounds are secondary metabolites of plants that have powerful antioxidant activity (Rajurkar and Gaikwad, 2012 ▶).  Free hydroxyl groups of phenolic compounds are essential for scavenging free oxygen radicals and producing anti-inflammatory effects leading to prevention of oxygen free-radicals release by leukocytes (Azuma et al., 1986 ▶). In agreement with our results, Nilforouzadeh et al. (2014) ▶ demonstrated that *A.* *capillus-veneris* had wound healing effects, probably due to angiogenic properties of polar components present in the aqueous extract and antioxidant activities of the plant flavonoids such as quercetin and kaempferol. Ishagh et al. (2014) ▶ and Pan et al. (2011) ▶ affirmed that antibacterial and antifungal effects of both ethanolic and aqueous extracts of *A.capillus-veneris* might be related to phenolic content of this plant. We know that a number of oral antibiotics and antiseptics such as metronidazole, ciprofloxacin and rifaximin are beneficial for IBD therapy especially when perianal fistula and abscesses became superimposed (Guslandi, 2011 ▶; Isaacs and Sartor, 2004 ▶). On the other hand, it was reported that *A.* *capillus-veneris* possesses antidiarrheal and antispasmodic effects through activation of ATP-dependent K^+^ channels present in intestinal smooth muscles (Janbaz et al., 2015 ▶). We know that diarrhea and bloody diarrhea, abdominal cramps/pains and bowel spasm are among symptoms of IBD for which, before-mentioned properties of *A.capillus-veneris* could be desirable (Head and Jurenka, 2003 ▶). *A. capillus-veneris* is rich in flavonoids and these compounds have antioxidant activities that protect the body from reactive oxygen species (ROS) (Tapas et al., 2008 ▶). Rutin and Isoquercetin are two important flavonoids of *A. capillus-veneris* that have antioxidant and free radical scavenging effects (Ghasemi-Dehkordi, 2002 ▶). They increase the level of glutathione in target cells which acts as a hydroxyl radical scavenger (Abdel‐Raheem, 2010 ▶; Bhatia et al., 2016 ▶). Rutin also inhibits neutrophil infiltration and reduces MPO activity. These properties were also supported by researchers who reported that rutin caused protection against indomethacin‐induced gastric ulcer in rats (Abdel‐Raheem, 2010 ▶). Mucilage is another compound that exists in *A. capillus-veneris* which could be accounted for anti-inflammatory, antioxidant and anti-ulcerative effects of this medicinal plant (Ghasemi-Dehkordi, 2002 ▶; Dugani et al., 2008 ▶; Sindhu et al., 2012 ▶). *Ocimum basilicum* Linn. seeds and *Aloe vera* gel are among mucilage-rich herbals exhibiting anti-colitic effects, likely through thickening and stabilizing the mucus membrane and improving antioxidant capacity of luminal cell lines (Saeidi et al., 2018 ▶; Park et al., 2011 ▶). So, it is assumed that mucilage content of *A. capillus-veneris* may play an important role in anti- ulcerative activities of this plant.

In conclusion, our research suggested that *A. capillus-veneris* had healing effects in ulcerative colitis, probably due to its phenolic compounds, flavonoids such as rutin, isoquercetin, triterpenoids and mucilage that express antioxidant, anti-inflammatory, and wound healing effects. Further experiments are required to explain exact mechanism of action of these extracts and their beneficial uses in clinical settings.
